# Obstructive sleep apnea therapy for cardiovascular risk reduction—Time for a rethink?

**DOI:** 10.1002/clc.23747

**Published:** 2021-11-17

**Authors:** Hasthi U. Dissanayake, Juliana T. Colpani, Kate Sutherland, Weiqiang Loke, Anna Mohammadieh, Yi‐Hui Ou, Philip de Chazal, Peter A. Cistulli, Chi‐Hang Lee

**Affiliations:** ^1^ Sleep Research Group, Charles Perkins Centre & Northern Clinical School, Faculty of Medicine and Health University of Sydney Camperdown New South Wales Australia; ^2^ Department of Cardiology National University Heart Centre Singapore Singapore; ^3^ Faculty of Dentistry National University of Singapore Singapore; ^4^ Department of Respiratory and Sleep Medicine Royal North Shore Hospital St Leonards New South Wales Australia; ^5^ Department of Medicine, Yong Loo Lin School of Medicine National University of Singapore Singapore; ^6^ Sleep Research Group, Charles Perkins Centre, Faculty of Engineering University of Sydney Camperdown New South Wales Australia

**Keywords:** blood pressure, cardiovascular risk, coronary artery disease, sleep apnea, upper airway

## Abstract

Obstructive sleep apnea (OSA) is a highly prevalent and underdiagnosed medical condition, which is associated with various cardiovascular and metabolic diseases. The current mainstay of therapy is continuous positive airway pressure (CPAP); however, CPAP is known to be poorly accepted and tolerated by patients. In randomized controlled trials evaluating CPAP in cardiovascular outcomes, the average usage was less than 3.5 hours, which is below the 4 hours per night recommended to achieve a clinical benefit. This low adherence may have resulted in poor effectiveness and failure to show cardiovascular risk reduction. The mandibular advancement device (MAD) is an intraoral device designed to advance the mandible during sleep. It functions primarily through alteration of the jaw and/or tongue position, which results in improved upper airway patency and reduced upper airway collapsibility. The MAD is an approved alternative therapy that has been consistently shown to be the preferred option by patients who are affected by OSA. Although the MAD is less efficacious than CPAP in abolishing apnea and hypopnea events in some patients, its greater usage results in comparable improvements in quality‐of‐life and cardiovascular measures, including blood pressure reduction. This review summarizes the impact of OSA on cardiovascular health, the limitations of CPAP, and the potential of OSA treatment using MADs in cardiovascular risk reduction.

**ABBREVIATIONS**
BP: blood pressure
CI: confidence interval
CPAP: continuous positive airway pressure
MAD: mandibular advancement device
OSA: obstructive sleep apnea

## 1 INTRODUCTION

Obstructive sleep apnea (OSA) is a sleep disorder characterized by repetitive upper airway collapse. In recent years, the prevalence of OSA has increased due to the obesity epidemic and population aging. It is estimated that up to 36% of the general population and 40%–80% of patients with cardiovascular disease have moderate‐to‐severe OSA (apnea‐hypopnea index ≥15 events/h).^1^, ^2^ OSA is an important public health challenge due to its association with excessive daytime sleepiness, motor vehicle accidents, and various manifestations of metabolic, cardiovascular, and cerebrovascular diseases.^3^, ^4^ Continuous positive airway pressure (CPAP), with positive airway pressure applied through a nasal or oronasal interface to splint the upper airway open, is the mainstay of therapy for OSA. CPAP is effective in ameliorating OSA‐associated sleepiness. Moreover, epidemiological data have demonstrated that patients with OSA who use CPAP have a lower risk of fatal and nonfatal cardiovascular events than nonusers.^5^ Similarly, randomized trials have shown the benefits of CPAP in improving surrogate markers such as blood pressure (BP), inflammation, and endothelial function.^6^ However, randomized controlled trials have failed to verify the benefits of CPAP in reducing cardiovascular events.^7^, ^8^, ^9^

**FIGURE 1:**
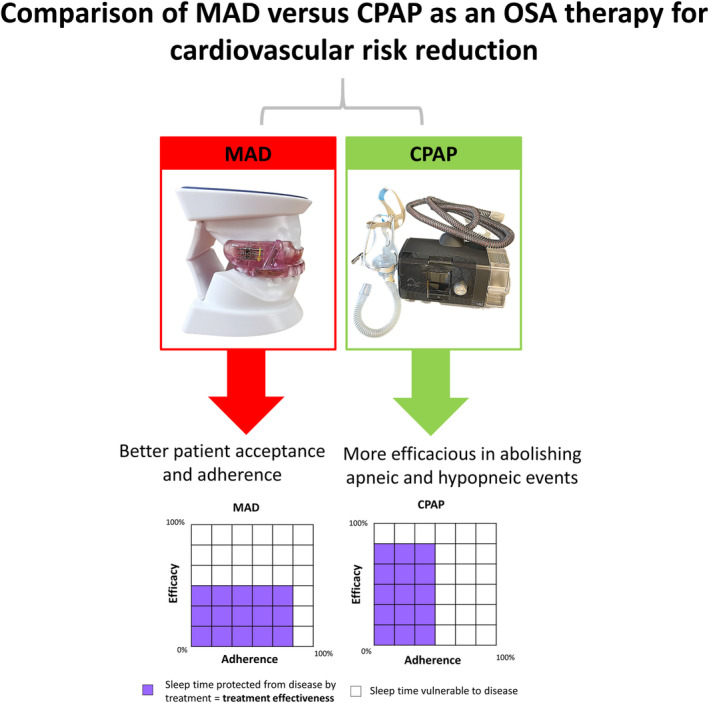
Brief history and comparison of MAD with CPAP. AASM, American Academy of Sleep Medicine, CPAP, continuous positive airway pressure, MAD, mandibular advancement device, OSA, obstructive sleep apnea

Various explanations for these conflicting findings have been proposed.^10^ These include poor CPAP adherence among trial participants who did not experience excessive daytime sleepiness, limited ability of the apnea–hypopnea index (the conventional measure of OSA severity) to risk‐stratify patients with OSA, and the trials being underpowered. Consequently, a panel discussion on long‐term outcome research in OSA was held during the SLEEP 2021 meeting, in which many suggestions were proposed; these included (i) adopting different study designs, (ii) replacing the apnea–hypopnea index with novel indexes to better identify at‐risk individuals, and (iii) exploring alternative therapies, such as a mandibular advancement device (MAD) (Figure 1). In this review article, we summarize the impact of OSA on cardiovascular disease and the potential of MADs in improving cardiovascular outcomes. Our narrative review complements recent systematic reviews and meta‐analyses and provides a comprehensive overview on MADs for cardiologists who do not have practical experience with the device.

## 2 OSA AND CARDIOVASCULAR STRESS

OSA is a complex and heterogeneous disease characterized by multiple underlying mechanisms (endotypes). The immediate effects of attempting to inspire against an obstructed upper airway include a drop in intrathoracic pressure, cortical arousal from sleep, hypoxia, and sympathetic activation.

Reduced intrathoracic pressure results in decreased left ventricular filling and increased afterload, ultimately reducing stroke volume. Furthermore, OSA causes marked, repeated BP elevation and tachycardia secondary to sympathetic nerve hyperactivity.^11^ The sympathetic nervous system is further augmented by decreased stroke volume and suppression of sympathetic inhibitory effects of lung stretch receptors during apnea. The net effect of increased left ventricular afterload, tachycardia, and BP elevation leads to myocardial oxygen supply–demand mismatch, ultimately resulting in (i) acute predisposition to cardiac ischemia and arrhythmias, and (ii) chronic predisposition to left atrial enlargement, left ventricular hypertrophy, and enlargement. Furthermore, recurrent upper airway collapse results in increased oxidative stress and reduced production of endothelium‐dependent vasodilator substances^12^ such as nitric oxide, which contribute to vascular dysfunction and systemic inflammation. These processes ultimately lead to myocardial fibrosis and left ventricular diastolic dysfunction. Sleep arousal and respiratory events during OSA increases sympathetic activity and peripheral vasoconstriction, and reduce parasympathetic modulation of the heart, resulting in elevated BP during the night. Hemodynamic consequences of OSA include oxidative stress, endothelial dysfunction, autonomic dysfunction, and catecholamine release. Collectively, the perturbations caused by OSA provide a clear mechanistic link to cardiovascular disease. OSA is strongly associated with hypertension, and a dose–response relationship exists between the severity of OSA and the degree of hypertension. In addition, OSA plays an important role in resistant hypertension and may mediate the association with cardiovascular disease.

## 3 CLINICAL TRIALS OF CPAP AND CARDIOVASCULAR OUTCOMES

Interest in the cardiovascular benefits of treating OSA was ignited by studies showing that OSA was an independent predictor of adverse cardiovascular events,^3^, ^13^ and that CPAP improved cardiovascular surrogate markers.^6^, ^14^ In the past decade, three randomized controlled trials have been conducted to explore the potential benefits of CPAP in cardiovascular risk reduction (Table 1). In the *randomized intervention with CPAP in CAD and OSA* (RICCADSA) trial,^8^ patients with newly revascularized coronary artery disease and OSA (apnea–hypopnea index ≥15 events/h) were randomized to CPAP (*n* = 122) or usual care (*n* = 122). The primary end point was a composite of repeat coronary revascularization, myocardial infarction, stroke, or cardiovascular mortality. Over a median follow‐up of 57 months, the incidence of the primary end point was 18.1% (CPAP group) versus 22.1% (usual care group, *p* = .449).

**TABLE 1:** Summary of the three randomized controlled trials on the effects of CPAP on cardiovascular events

Trials	Single or multicenter study	Number of patients recruited	Study period	Key inclusion criteria	Key exclusion criteria	Definition of OSA	Mean ESS (CPAP vs. usual care)	Average CPAP adherence (h/night)	Percentage of patients with adherence ≥4 h/night)
RICCADSA	Single‐center	244	2005–13	Adult patients with CAD who had undergone PCI or CABG in the previous 6 months	Patients with existing OSA, daytime sleepiness (ESS >10), and predominantly central apneas with Cheyne‐Stokes Respiration	AHI >15 events/h	NA	NA	NA
SAVE	Multicenter	2717	2008–16	Adults between 45 and 75 years of age who had OSA and stable coronary or cerebrovascular disease	Severe daytime sleepiness (ESS >15) or were considered to have an increased risk of an accident from falling asleep, very severe hypoxemia, or Cheyne‐Stokes Respiration	ODI ≥12 events/h	7.3 ± 3.6 versus 7.5 ± 3.6	3.3 h/night	42%
ISAACC	Multicenter	2551	2011–18	Aged ≥18 years, hospitalized for ACS	Previous treatment with CPAP for OSA, inability to complete questionnaires, known sleep disorder, >50% central apneas or the presence of Cheyne‐Stokes Respiration, and daytime sleepiness (ESS >10)	AHI ≥15 events/h	5.4 ± 2.5 versus 5.3 ± 2.5	2.8 h/night	38%

Abbreviations: ACS, acute coronary syndrome; AHI, apnea‐hypopnea index; CABG, coronary artery bypass grafting; CAD, coronary artery disease; CPAP, continuous positive airway pressure; ESS, Epworth Sleepiness Scale (scores range from 0 to 24, with higher scores indicating greater severity. Daytime sleepiness generally defined as ESS >10), NA, not available; ODI, Oxygen desaturation index (the number of times per hour during the oximetry recording that the blood oxygen saturation level drops by ≥4 percentage points from baseline); OSA, obstructive sleep apnea; PCI, percutaneous coronary intervention.

The *sleep apnea cardiovascular endpoints* (SAVE) trial^7^ was the largest multicenter randomized trial conducted to date. Patients with stable coronary artery disease or cerebrovascular disease (*n* = 2717) and OSA (oxygen desaturation index ≥12 events/h) were randomized to CPAP (*n* = 1346) or usual care (*n* = 1341). The primary outcome was a composite of death from cardiovascular causes, myocardial infarction, stroke or hospitalization for unstable angina, and heart failure or transient ischemic attack. Over a mean follow‐up of 3.7 years, the incidence of the primary end point was 17.0% (CPAP group) versus 15.4% (usual care group, *p* = .34).

The *CPAP in patients with acute coronary syndrome and OSA* (ISAACC) trial^9^ was a multicenter randomized trial of patients with the acute coronary syndrome. All patients underwent respiratory polygraphy during the acute phase, and patients with OSA were randomized to CPAP (*n* = 633) or usual care (*n* = 631). The primary end point was a composite of cardiovascular events (cardiovascular death or nonfatal events [acute myocardial infarction, nonfatal stroke, hospital admission for heart failure, and new hospitalizations for unstable angina or transient ischemic attack]). Over a median follow‐up of 3.4 years, the incidence of the primary end point was 16% (CPAP group) versus 17% (usual care group, *p* = .40).

Superficially, all three trials showed no clear benefit of OSA therapy using CPAP in improving cardiovascular outcomes. However, further data analysis revealed limitations in the design and execution of the studies. The adherence of CPAP in these trials was low. Indeed, none of these trials reported an average CPAP adherence of ≥4 h/night, which is the minimum adherence needed to derive benefits from CPAP.^15^ Notably, these trials recruited patients who had developed cardiovascular events. Such patients are different from younger patients whom CPAP may confer a benefit in primary prevention of cardiovascular disease, as they may be more likely to be adherent to CPAP.

## 4 LOW CPAP ADHERENCE IN CLINICAL TRIALS AND ITS IMPLICATIONS

Although CPAP is the guideline‐mandated first‐line treatment for OSA, it has long been recognized that some patients do not accept the therapy or give up after the first few weeks. Using contemporary data from 789 260 patients initiated on CPAP in the US Centers of Medicare & Medicaid Services database, the overall adherence (≥ 4 hours of use on 70% of nights over a consecutive 30‐day period) in the first 90 days was only 72.6%.^16^ Although these patients presented with symptomatic OSA, a visible decrease in use over time was observed.^16^ To rectify this problem, interventions such as mask optimization, heated humidification, topical nasal therapy, education programs, and patient engagement apps have been introduced.^17^ However, these interventions have had limited success, and this treatment modality continues to be plagued by problems with adherence. Indeed, the overall nonadherence remains consistent at 30%–40%, especially in health systems where the cost of CPAP is not reimbursable.^18^, ^19^, ^20^

Given the above, it is not surprising that many research participants with cardiovascular disease, who tend to be less sleepy, were unable to tolerate the CPAP over the duration of the trials to achieve clinically significant benefits. In the RICCADSA trial, 38% of the participants in the CPAP group stopped using the device within the first year. The adjusted on‐treatment analysis showed a cardiovascular risk reduction in those who used CPAP for ≥4 versus <4 h/night (*p* = .026), suggesting that the low CPAP adherence may have contributed to the overall negative results. In the SAVE trial, despite the initial run‐in period with sham CPAP achieving an average usage of 5.2 h/night, CPAP usage declined over the first year to 3.5 ± 2.4 h/night, and was only 3.3 ± 2.3 h/night at the final follow‐up. Moreover, only 42% of participants in the CPAP group achieved the conventional criteria for good adherence (≥ 4 h/night). The propensity score‐matched analyses showed that the patients who were adherent to CPAP therapy had a lower risk of stroke (*p* = .05) and composite end point of cerebral events (*p* = .02) than those in the usual care group. Similarly, the adherence to CPAP was extremely low in the ISAACC study. Indeed, 1 year after starting CPAP, the average adherence was only 2.8 ± 2.6 h/night, with only 36% of the patients in the CPAP group achieving ≥4 h/night. A propensity score analysis comparing patients who achieved “good adherence” with those receiving usual care showed a hazard ratio of 0.80 favoring the CPAP group. However, the small sample size precluded a demonstration of statistical significance.

## 5 MANDIBULAR ADVANCEMENT DEVICE—AN ALTERNATIVE TO CPAP FOR CARDIOVASCULAR RISK REDUCTION

**FIGURE 2:**
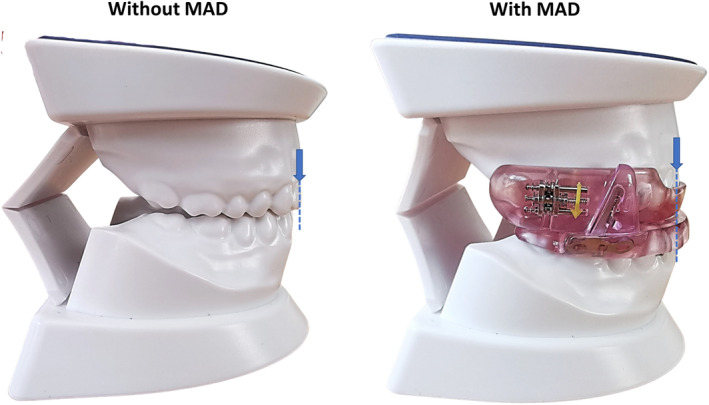
A MAD functions primarily through protrusion of the lower jaw (blue arrows). The amount of protrusion is titratable based on patient's tolerance. MAD, mandibular advancement device

MADs offer a viable alternative to CPAP owing to the noninvasive nature of the therapy and higher acceptance among patients with OSA. The impact of tongue and mandibular positioning on upper airway patency has been well known for over 100 years and remains the functional basis of the “jaw thrust” during cardiopulmonary resuscitation. MADs are intraoral devices that are designed to advance the mandible during sleep. They function primarily through alteration of the jaw and/or tongue position (Figure 2), which results in improved upper airway patency/anatomy and reduced upper airway collapsibility. In its latest 2015 guidelines, the American Academy of Sleep Medicine recommends MADs for patients with primary snoring, mild‐to‐moderate OSA, and in adult patients with OSA who are intolerant of CPAP therapy or prefer alternative therapy.^21^

When a MAD is prescribed for a patient with OSA, it is recommended that a qualified dentist uses a custom‐made, titratable device rather than non‐custom oral devices. It is imperative to note that even though MADs are primarily administered by dentists, OSA should be treated as a chronic disease entity requiring long‐term, multidisciplinary management, and follow‐up.

### 5.1 Mechanisms of action

**FIGURE 3:**
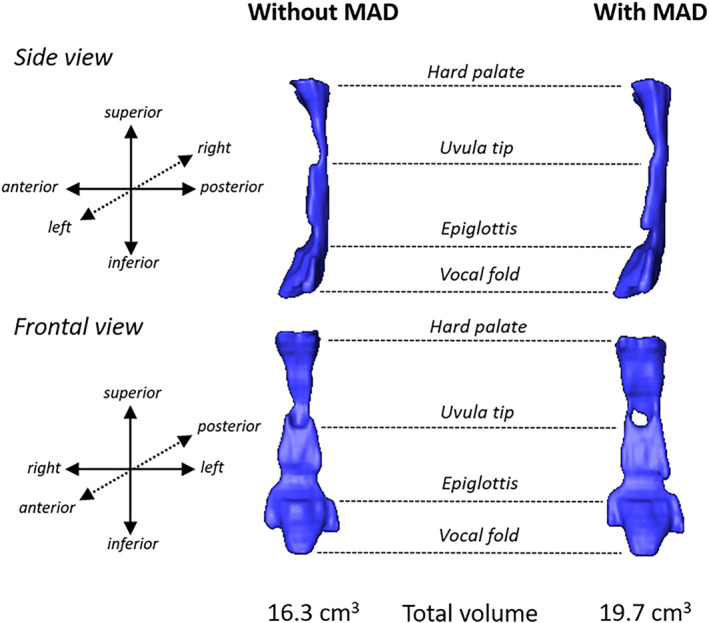
Volumetric reconstructions of the upper airway space constructed from Magnetic Resonance Imaging (MRI) scans in a MAD treatment responder without and with a MAD in situ. The anatomical landmarks are shown from the hard palate at the top to the vocal folds at the bottom. The reconstructed airway is shown from the side view (sagittal plane) and frontal view (coronal plane). An increase in the airway space can be observed with MAD, particularly in the frontal view where a widening in the coronal plan can be observed at various points along the upper airway. Often this lateral widening is most prominent in the region between the hard palate and uvula tip. The total volume of the airway increased from 16.3 to 19.7 cm^3^ with MAD. The apnea‐hypopnea index decreased from 42.8 to 10.7 events/h with MAD. MAD, mandibular advancement device

Magnetic resonance imaging studies show that MADs act to enlarge the upper airway space, most notably in the lateral dimension of the velopharyngeal region (Figure 3).^22^ This lateral expansion of the airway space is probably mediated through lateral tissue movement via direct tissue connections between the pharyngeal lateral walls and the ramus of the mandible. Various patterns of anterior tongue movement have also been observed to occur with mandibular advancement. These patterns include en bloc anterior movement, superior–inferior compression, and anterior movement associated with the posterior nasopharyngeal and oropharyngeal regions of the tongue, which may lead to different amounts of efficacy.

Physiologic studies have further demonstrated that MADs primarily act on the reduction of upper airway anatomy and collapsibility under both passive and active conditions, in a dose‐dependent fashion. MADs achieve this without affecting nonanatomical contributors to OSA, including upper airway dilator muscle compensation or ventilatory control mechanisms. It has also been observed that patients with a mild anatomic compromise and lower loop gain measurements at baseline tend to show a greater response to therapy. Additionally, a study based on computational fluid dynamic simulation found that after the MAD intervention, the narrowest area of the upper airway was located in the lower bound of the velopharynx, where the volume and pressure were significantly increased (*p* < .05) and the air velocity was significantly decreased from baseline (*p* < .05). Pharyngeal airflow resistance was also reduced by 35.9%.^23^

### 5.2 Design

There is currently no conclusive evidence to indicate that a specific MAD design is most effective in improving polysomnographic indices. The efficacy of a MAD depends on many factors, including the severity of OSA, materials, and methods of fabrication, type of MAD (monobloc/twin block), and the degree of protrusion (sagittal or vertical). Nevertheless, customized devices are preferred over thermoplastic devices as they are associated with a higher rate of treatment success (60% customized vs. 31% non‐customized). Lower adherence of thermoplastic devices has also been shown, which is attributable to insufficient retention of the device during sleep.

### 5.3 Adherence

Using temperature microsensors to collect objective adherence data is a major advance in evaluating OSA therapy using MADs. Most published studies have reported a daily adherence of >6 h/night in the early phase of treatment.^24^ The *ORM Narval mandibular repositioning device in the second‐line treatment of severe OSAH obstructive sleep apnea–hypopnea* (ORCADES) trial was a French multicenter cohort study that investigated the long‐term effectiveness of MAD therapy in patients with OSA who refused or were intolerant of CPAP. Among the 369 recruited participants, 92% and 75% remained on the therapy at the 6‐month and 2‐year follow‐ups, respectively. At the 5‐year follow‐up, 96.5% of the patients reported that they wished to continue the MAD therapy.^25^

### 5.4 Therapeutic efficacy

In the past decade, several randomized controlled trials evaluating the effect of MAD on the apnea–hypopnea index have been published, including studies comparing MAD and CPAP. Thirty‐four randomized controlled trials with 1301 patients assessed the effect of MAD on the apnea–hypopnea index and found an overall improvement. A meta‐analysis was performed on all included trials that compared the apnea–hypopnea index pre‐ and posttreatment with MAD.^26^ In weighted analysis, the mean reduction in the apnea–hypopnea index was 13.6 events/h (95% CI: −15.3, −12.0) with a MAD relative to the control group without MAD. Twenty‐five of the 34 randomized controlled trials included in the meta‐analysis reported a >50% reduction in the apnea–hypopnea index with the use of MADs in adult patients with OSA. It cannot be overemphasized that, similar to CPAP, the benefit of MAD is also related to device adherence. Recent data highlight variable MAD usage patterns across patients, and further research is needed to understand the basis of this and methods of improving adherence.^26^

### 5.5 Oral health and MADs

The importance of good oral health cannot be overemphasized. There is an intricate relationship between oral health, cardiovascular disease, and OSA therapy using MADs. Edentulism, a marker of poor oral health, increases the risk of OSA by 2% for each additional lost tooth among adults aged 25–65 years, with a dose‐dependent association.^27^ Compared with adults with 0–4 missing teeth, the OSA risk is 25% greater when adults have 5–8 teeth missing, 36% greater when 9–31 teeth are missing, and 61% greater in the completely edentulous. Of even greater significance, edentulism is associated with cardiovascular health and is an independent risk factor for cardiovascular events, with each missing tooth associated with a 1% increase in myocardial infarction, a 1.5% increase in heart failure and stroke, and a 2% increase in mortality.^28^ Whenever a MAD is prescribed, the success of the device often depends on the oral health of patients. Edentulism considerably reduces the suitability of MAD as sufficient dentition is necessary to support and retain the device. Besides edentulism, other dental contraindications that preclude the use of MAD may include periodontal problems such as tooth mobility, active temporomandibular joint disorder, and limited maximum protrusive distance (<6 mm).

### 5.6 Side effects of MADs

A recent meta‐analysis found a significant change in overbite and overjet with MAD use. However, their extent and importance remain unclear and need to be considered against the benefits of treating OSA. Another limiting factor in MAD therapy is patient preference. Some patients cannot tolerate wearing a MAD. Commonly reported problems include the device falling out overnight, oral dryness, excessive saliva production, and masticatory muscle discomfort. Furthermore, it is known that poor dental health is associated with cardiovascular disease and poor dental health may be a contraindication for the use of MAD. This could potential limit the role of MAD in some patients with cardiovascular disease.^29^

### 5.7 Tongue retaining devices

Although this review focuses on MADs, studies involving tongue retaining devices have also shown efficacy in the management of OSA. A systematic review demonstrated tongue retaining devices having a substantial effect (a relative reduction of apnea–hypopnea index by 53% and oxygen desaturation index by 56%) from baseline, and may provide an effective alternative treatment option for OSA. It is important to note that these are results garnered from supervised studies as opposed to over‐the‐counter tongue devices that were not selected and delivered by dentists.^30^, ^31^

## 6 EFFICACY VERSUS EFFECTIVENESS OF OSA TREATMENT

In comparing the use of a MAD and CPAP for OSA therapy, the difference between the efficacy and effectiveness is of paramount importance. In this context, efficacy refers to the *ability* of the treatment to prevent the occurrence of obstructive breathing events when the treatment is physically applied. Effectiveness refers to the degree to which CPAP is successful in producing the desired result when prescribed to patients, and encompasses both efficacy and adherence. Only in patients who are fully adherent to the prescribed treatment is effectiveness the same as efficacy. In this regard, CPAP is notorious for its poor acceptance and adherence, as most patients only apply it for approximately half of their sleeping time (frequently the first half).^32^ Considering OSA recurrence during the untreated half of the night, the effectiveness of CPAP is <50% of its efficacy. This has not been considered in the cardiometabolic stress of rapid eye movement sleep, which occurs during the second half of sleep, frequently after patients have removed the CPAP. Hence, the clinical effectiveness of CPAP is undermined by patient intolerance. In contrast, although MADs may be less efficacious than CPAP in some patients when applied for the same duration, they are both well tolerated and preferred by patients. It was reported that 81% of patients preferred MADs at the end of a cross‐over trial of MADs and CPAP.^33^ Moreover, in a randomized trial comparing a MAD and CPAP, greater adherence to the MAD was consistently observed in patients with mild, moderate, and severe OSA. The treatment preference results showed that 51% preferred the MAD, 23% preferred CPAP, 21% preferred either, and 5% preferred no treatment.^34^ Therefore, MADs have the potential to offer greater treatment effectiveness in reducing OSA in cardiovascular research trials.

## 7 MADs AND CARDIOVASCULAR RISK

Although CPAP is more effective than MADs in reducing the apnea–hypopnea index,^35^ there is a growing body of evidence demonstrating comparable benefits between MADs and CPAP in ameliorating OSA‐associated quality of health and adverse health consequences.^36^

### 7.1 MADs and BP reduction

The relationship between OSA and hypertension is best evidenced by the treatment of OSA using CPAP, which works by lowering incident hypertension^37^ and BP in patients with pre‐existing hypertension.^38^ Many clinical trials have shown similar effectiveness between MADs and CPAP in terms of BP reduction.^39^, ^40^ As the sample sizes of all these trials were less than 150 participants, systematic review and meta‐analysis play an important role in providing insight into the effects of MAD on BP reduction. It is worthwhile to note that these data comprised a wide range of OSA severity, some included patients with mild to moderate OSA, and some had severe OSA patients.

In the largest meta‐analysis so far, Pengo et al. included 68 randomized controlled trials that compared CPAP or MADs with either passive or active treatment.^41^ Overall, both the CPAP and MADs were associated with BP reduction. CPAP was associated with an average BP reduction when compared with passive treatment of −2.09 (95% confidence interval [CI] −2.78 to −1.40) mm Hg for systolic BP and −1.92 (95% CI −2.40 to −1.43) mm Hg for diastolic BP. Similar results were reported considering MAD treatment versus passive control with a systolic BP change of −1.27 (95% CI −2.34 to −0.20) mm Hg and a diastolic BP change of −1.11 (95% CI −1.82 to −0.41) mm Hg. There was no significant difference between CPAP and MADs in their association with the change in systolic BP (0.26 [95% CI −1.07 to 1.60] mm Hg) or diastolic BP (0.15 [95% CI −0.58 to 0.89] mm Hg).

Bratton et al. included 5151 randomized controlled trials (4888 patients) in the analysis; these included 44 that compared CPAP with an inactive control, 3 that compared MADs with an inactive control, one that compared CPAP with a MAD, and three that compared CPAP, a MAD, and an inactive control. Compared with the inactive control, CPAP was associated with a reduction in systolic and diastolic BP of 2.5 mm Hg (*p* < .001) and 2.0 mm Hg (*p* < .001), respectively. A 1‐hour‐per‐night increase in mean CPAP use was associated with an additional reduction in systolic BP of 1.5 mm Hg (*p* < .001) and an additional reduction in diastolic BP of 0.9 mm Hg (*p* = .001). Compared with the inactive control, the use of a MAD was associated with a reduction in systolic and diastolic BP of 2.1 mm Hg (*p* = .002) and 1.9 mm Hg (*p* = .008), respectively. There was no significant difference between CPAP and the MAD in terms of the change in systolic BP (*p* = .55) or diastolic BP (*p* = .82).^42^

De Vries et al. published a meta‐analysis including 16 articles (11 randomized controlled trials) on OSA therapy using a MAD alone, or a MAD compared with another treatment (placebo, CPAP, lifestyle intervention, surgery). Among the eight studies that reported BP variables (all without a surgery arm), the results showed a significant reduction in both daytime systolic BP (−1.8 mm Hg, *p* < .05) and daytime diastolic BP (−2.2 mm Hg, *p* = .009) compared with baseline values.^43^ Compared to CPAP therapy, the MAD was found to be equally effective in reducing BP (mean difference in change for systolic BP: 0.05 mm Hg, *p* = .98, and for diastolic BP: 0.23 mm Hg, *p* = .81). Yet, the reduction in BP with MAD compared to inactive controls (inactive/placebo oral appliance, conservative measures) did not reach statistical significance (mean change −1.55 mm Hg [95% CI −3.92 to 0.82], *p* = .20, and mean change −1.14 mm Hg [95% CI −2.87 to 0.59], *p* = .20 for systolic and diastolic BP, respectively.

### 7.2 MADs and other cardiovascular end points

To date, there is a relative paucity of large‐scale randomized clinical trials on the effects of MADs, and the relative effectiveness of CPAP and MADs with other cardiovascular end points, including atrial fibrillation and heart failure. In a cohort study with long‐term follow‐up, 208 control subjects without OSA, 177 patients with OSA treated with CPAP, 72 with a MAD, and 212 who declined treatment were analyzed. Forty‐two patients had cardiovascular death during the median follow‐up of 6.5 years. The non‐OSA group had the lowest cardiovascular death rate (0.28 per 100 person‐years), followed by the CPAP‐treated (0.56 per 100 person‐years) and the MAD‐treated OSA groups (0.61 per 100 person‐years), with the highest cardiovascular mortality rate observed in the untreated OSA group (2.1 per 100 person‐years). There was no significant difference in cardiovascular mortality between the CPAP and MAD groups (hazard ratio: 1.08, *p* = .71).^44^

## 8 WEIGHT REDUCTION AND OSA

By correcting obesity—a major risk factor for OSA, weight reduction has been explored as a standalone or adjunctive therapy for OSA therapy. In the Wisconsin Sleep Study,^45^ a weight loss of 10% translated into a 26% decrease in apnea‐hypopnea index. In a meta‐analysis that included seven randomized controlled trials and three nonrandomized studies), one unit of body mass index reduction was found to be associated with changes in the apnea‐hypopnea index (−2.83/h; 95% CI: −4.24, −1.41), systolic BP (−1.86 mm Hg; 95% CI: −3.57, −0.15) and diastolic BP (−2.07 mm Hg; 95% CI: −3.79, −0.35). It is conceivable that weight reduction (via exercise and reduced calorie intake) and MAD therapy will provide a superior effect in BP reduction than either therapeutic approach alone.^46^ In a randomized trial of patients with OSA, systolic BP was reduced at 24 weeks in patients treated with CPAP, weight reduction, and combined intervention, with no significant between‐group differences. In a secondary analysis, adherence to a regimen of weight loss and CPAP may result in incremental reductions in BP as compared with either intervention alone.^47^

## 9 FUTURE PERSPECTIVES

Over the past two decades, there has been a plethora of research on OSA and cardiovascular disease. Currently, OSA is recognized as a cardiovascular risk factor. The repeated failure to demonstrate the cardiovascular benefits of CPAP due to poor adherence, and the emergence of MADs as a viable alternative, suggest it is time for the medical community to reconsider the relative merits of CPAP and MADs. So far, most of the studies on the effects of MADs on cardiovascular risk have been small‐scale and have focused on BP reduction. We believe that efforts and resources to thoroughly evaluate the potential of MADs in improving cardiovascular outcomes are urgently needed.

As highlighted by the American Heart Association Scientific Statement on OSA and cardiovascular disease,^2^ further research is also needed to ascertain which subgroups with OSA and cardiovascular disease will benefit most from MADs. Notably, there is a need to better understand the eligibility, acceptance, and adherence of MADs in patients with cardiovascular diseases. It is conceivable that big‐data methods, such as multi‐omics profiling, could identify novel disease mediators as potential diagnostic and therapeutic targets for OSA.

Evidence from a growing number of multiethnic population studies suggests that OSA is more prevalent and severe among East Asians (Chinese, Japanese, and Koreans) than those of European and Indian ancestries.^48^, ^49^ While obesity is the strongest contributing risk factor for OSA in all ethnic groups studied thus far, OSA occurs at a lower body mass index in East Asians. This may reflect a stronger influence of craniofacial restriction on upper airway size in East Asians. Therefore, as a therapy targeting craniofacial restriction, MADs may be particularly effective in East Asians with OSA.

## 10 CONCLUSION

OSA is a prevalent and treatable sleep disorder associated with fatal and nonfatal cardiovascular events. Despite its efficacy in restoring upper airway patency and ventilation during sleep, CPAP remains an imperfect modality of treatment. Patient acceptability and adherence are a challenge, especially in cardiovascular patients, many of whom do not exhibit excessive daytime sleepiness. MADs are better tolerated than CPAP, and have recently emerged as a viable alternative for OSA. While thorough evaluation of the role MADs in cardiovascular risk reduction evaluated by randomized controlled trials is at an early stage, the potential benefits of MAD are immense. In the meantime, clinicians should preferentially use MADs for patients with approved indications and those who have refused CPAP or prefer MADs.

## CONFLICT OF INTEREST

Anna Mohammadieh has received in‐kind support from Resmed Pte Ltd and SomnoMed Australia Ltd for investigator‐led studies. Kate Sutherland has received in‐kind support from SomnoMed Australia Ltd. Peter A. Cistulli has an appointment as an endowed Academic Chair at the University of Sydney that was created from ResMed funding; he receives no personal fees, and this relationship is managed by an Oversight Committee of the University. Additionally, he has received research support from ResMed, SomnoMed, Zephyr Sleep Technologies, and Bayer, and is a consultant/adviser to Zephyr Sleep Technologies, Signifier Medical Technologies, SomnoMed, ResMed, and Bayer. He also has a pecuniary interest in SomnoMed related to a previous role in R&D (2004). Philip de Chazal holds an endowed Academic Chair at the University of Sydney, which was established through funding from ResMed and has received research support from ResMed and SpaceLabs. Chi‐Hang Lee has received research grant from Boston Scientific Corporation.

## Data Availability

Data sharing not applicable ‐ no new data generated. This is a review article.
